# Genetic and environmental sources of familial coaggregation of obsessive−compulsive disorder and suicidal behavior: a population-based birth cohort and family study

**DOI:** 10.1038/s41380-019-0417-1

**Published:** 2019-04-08

**Authors:** Anna Sidorchuk, Ralf Kuja-Halkola, Bo Runeson, Paul Lichtenstein, Henrik Larsson, Christian Rück, Brian M D’Onofrio, David Mataix-Cols, Lorena Fernández de la Cruz

**Affiliations:** 1 Centre for Psychiatry Research, Department of Clinical Neuroscience, Karolinska Institutet, & Stockholm Health Care Services, Stockholm County Council, Stockholm, Sweden; 2 Department of Medical Epidemiology and Biostatistics, Karolinska Institutet, Stockholm, Sweden; 3 Centre for Psychiatry Research, Department of Clinical Neuroscience, Karolinska Institutet, & Stockholm Health Care Services, Stockholm County Council, S:t Görans Hospital, SE-112 61 Stockholm, Sweden; 4 School of Medical Sciences, Örebro University, Örebro, Sweden; 5 Department of Psychological and Brain Science, Indiana University, Bloomington, IN USA

**Keywords:** Diseases, Psychiatric disorders

## Abstract

Obsessive−compulsive disorder (OCD) is associated with high risk of suicide. It is yet unknown whether OCD and suicidal behaviors coaggregate in families and, if so, what are the mechanisms underlying this coaggregation. In a population-based birth cohort and family study, we linked individuals born in Sweden in 1967–2003 (*n* = 3,594,181) to their parents, siblings, and cousins, and collected register-based diagnoses of OCD, suicide attempts, and deaths by suicide and followed them until December 31, 2013. We also applied quantitative genetic modeling to estimate the contribution of genetic and environmental factors to the familial coaggregation of OCD and suicidal behavior. An elevated risk of suicide attempts was observed across all relatives of individuals with OCD, increasing proportionally to the degree of genetic relatedness, with odds ratios (OR) ranging from 1.56 (95% confidence interval (CI) 1.49–1.63) in parents to 1.11 (95% CI 1.07–1.16) in cousins. The risk of death by suicide also increased alongside narrowing genetic distance, but was only significant in parents (OR 1.55; 95% CI 1.40–1.72) and full siblings (OR 1.80; 95% CI 1.43–2.26) of individuals with OCD. Familial coaggregation of OCD and suicide attempts was explained by additive genetic factors (60.7%) and non-shared environment (40.4%), with negligible contribution of shared environment. Similarly, familial coaggregation with death by suicide was attributed to additive genetics (65.8%) and nonshared environment (34.2%). Collectively, these observations indicate that OCD and suicidal behaviors coaggregate in families largely due to genetic factors. The contribution of unique environment is also considerable, providing opportunities to target high-risk groups for prevention and treatment.

## Introduction

Recent evidence suggests that obsessive−compulsive disorder (OCD) is associated with a substantial risk of suicidal behavior, including high rates of suicidal attempts and death by suicide [1–5]. However, the mechanisms that may underlie the association between OCD and suicidal behaviors remain to be explored.

Both OCD and suicidal behaviors aggregate in families. Family and twin studies suggest that susceptibility to OCD is due to both genetic and nonshared environmental factors in equal proportion, while the contribution of shared environment seems to be negligible [6–8]. The familial clustering of suicide has shown to be primarily influenced by genetic and also shared environmental factors [9–13]. One yet unexplored possibility is that OCD and suicidal behaviors might share genetic and/or environmental risk factors, as shown in other psychiatric disorders. For example, a substantial contribution of shared genetic factors is reported for coaggregation of suicidal behavior and eating disorders [14], while shared genetic factors along with limited but plausible influence of familial environment are suggested to explain a coaggregation of attention-deficit/hyperactivity disorder with suicide attempts [15]. The study of such etiological aspects may contribute to the development of more effective ways of identifying and modifying the risk of suicide in OCD, particularly if the familial coaggregation between OCD and suicidal behavior is not entirely explained by genetic factors.

This population-based study of over 3 million Swedes examined whether OCD and suicidal behaviors coaggregate in families, and employed quantitative genetic modeling methods to estimate the contribution of genetic and environmental factors to such familial coaggregation.

## Methods

The study was approved by the Regional Ethics Review Board in Stockholm, Sweden (Reference number 2013/862-31/5). The requirement for informed consent was waived because the study was register-based and the included individuals were not identifiable at any time.

### Swedish national registers and study population

We conducted a population-based birth cohort and family study by linking the following nationwide Swedish registers via the unique personal identification number assigned to all Swedish residents [16]: the Multi-Generation Register (MGR) [17], the National Patient Register (NPR) [18], the Cause of Death Register (CDR) [19], the Total Population Register (TPR), and the Migration Register [20]. Details about these registers are given in the Supplementary information.

The study population consisted of all individuals born in singleton births in Sweden between January 1, 1967 and December 31, 2003 with information on both biological parents (*n* = 3,710,392). We excluded individuals who were adopted or had died or emigrated from Sweden before age 6 years. The final cohort included 3,594,181 individuals who were followed up until December 31, 2013.

### Family-level data

For each cohort member, five clusters of biological relatives were identified via the MGR: parents, full siblings (defined if they shared both parents), maternal half siblings (if they shared the same mother), paternal half siblings (if they shared the same father), and full cousins (if they shared a grandmother and grandfather). Siblings and cousins were nested in the final cohort. Parents were excluded from the analysis of parental cluster if one or both died or emigrated from Sweden before 1973 (i.e., before the full coverage of psychiatric disorders in the NPR) and did not return. Each person could appear multiple times in different clusters (e.g., parent, sibling, and cousin) and several relative pairs could be descended from one individual (e.g., in case of having several siblings). In each pair, we defined the person used for establishing the family relationship as the “proband”, with parents, siblings or cousins defined as “relatives”. Family identification numbers were created to link the relatives.

### Measures

Lifetime OCD diagnoses were retrieved from the NPR based on International Classification of Disorders (ICD) codes (see Supplementary Table 1). The ICD-10 codes for OCD have shown high validity, with moderate validity for ICD-8 and ICD-9 [21]. To avoid diagnostic misclassification, diagnoses were collected if recorded at age 6 years or above.

Suicide attempts with certain or undetermined intent were also identified from the NPR based on ICD-8/9/10 codes (see Supplementary Table 1) [2, 22]. Deaths by suicide were identified via the CDR as any records of suicide or external cause of death with undetermined intent by the same ICD codes (see Supplementary Table 1). Undetermined attempts and deaths were included to reduce underestimation of suicidal behavior [23–25]. Data on suicide attempts or deaths by suicide were collected if recorded at age 10 years or above to avoid inclusion of misclassified events [22, 26].

We collected data on lifetime psychiatric comorbidities given their known impact on suicidal behavior [27, 28]. The ICD codes for affective disorders, anxiety disorders, personality disorders, substance use disorders, psychotic disorders, and “other” psychiatric disorders, including reaction for severe stress, adjustment, dissociative, somatoform and other neurotic disorders, were retrieved from the NPR, if recorded at age 6 years or above (see Supplementary Table 1). Sex and year of birth (continuous variable, further categorized by 10-year increments) were retrieved from the TPR.

### Statistical analysis

Differences in clinical characteristics by OCD status were ascertained for the whole cohort and within gender strata with chi-square test and Fisher’s exact test for categorical variables and independent-sample two-tailed *t* tests for continuous variables. We quantified the association of OCD with suicide attempts and, separately, deaths by suicide in the total cohort and in each relative cluster, using logistic regression to obtain odds ratio (ORs) and corresponding 95% confidence intervals (CIs) [29]. All analyses were clustered by family identification number and a robust sandwich estimator of standard errors was used to account for nonindependence between repeated observations within families [30, 31].

In the total cohort, we compared the odds of having suicide outcomes in individuals with OCD to the odds in their counterparts without OCD in a model adjusted for sex and year of birth (categorical). Interactions between OCD and sex were detected in association with suicide attempts (*p* < 0.001) and death by suicide (*p* = 0.001) and therefore the results are presented separately for males and females. No significant interactions with sex of either probands or relatives appeared in the analyses of relative clusters.

In each relative cluster, we assessed the odds of attempting suicide and dying by suicide in relatives of OCD-probands in comparison to relatives of unaffected individuals, controlling for sex and year of birth (categorical) of both probands and relatives (only for proband’s sex and year of birth in parental cluster). Higher odds of suicide outcomes in relatives of OCD-probands indicate the contribution of shared genetics and environment to familial coaggregation of the disorders. The following assumptions on the source of coaggregation were made: (i) parents share 50% of additive genetic factors with offspring and provide the early rearing environment; (ii) full siblings share on average 50% of additive genetic factors and 100% of environmental factors; (iii) maternal half siblings share on average 25% of genetic factors and 100% of environmental factors; (iv) paternal half siblings share on average 25% of genetic factors and less than 100% of environmental factors; and (v) full cousins share on average 12.5% of genetic factors and 0% of environment [32]. The proportions of shared environment have been suggested since in Sweden children predominantly reside with mothers after parental separation and cousins rarely reside together [33].

The importance of genetic factors in familial coaggregation is suggested if significant association is observed in full cousins or if an association among full siblings is stronger than that in maternal half siblings. A contribution of shared environment is inferred if an association is stronger in maternal than in paternal half siblings. No difference between maternal and paternal half siblings in combination with significantly stronger association in full siblings additionally indicates the importance of genetic factors. In our study, we focused specifically on singletons due to their genuine difference with twins in intrauterine and perinatal conditions, as these conditions have  shown to impact the risk of OCD [34] and suicidal behaviors [35]. Furthermore, from full cousins’ cluster we excluded individuals whose parents were twins because the offspring of monozygotic twins share 25% of genetic factors. Table 1 presents the total number of individuals in the analyses.

**Table 1:** Number of individuals in the study cohort and each cluster of relatives

Total cohort and family clusters	Unique individuals	Unique pairs^a^	Observations^b^	Excluded
Total cohort	3,594,181	NA	3,594,181	NA
Parents−offspring	3,560,020	3,560,020	3,560,020	34,161^c^
Full siblings	2,726,045	2,071,762	4,143,524	868,136^d^
Maternal half siblings	471,824	357,365	714,730	3,122,357^d^
Paternal half siblings	512,484	416,289	832,578	3,081,697^d^
Full cousins	2,358,636	6,917,441	13,834,882	1,235,545^e^

Note: In all pairs of siblings and cousins, each individual contributed to the analysis, at least once, with information on exposure (OCD) and on outcome (suicide outcomes). Parents were measured on their suicide outcomes and offspring on OCD status

*NA* not applicable, *OCD* obsessive−compulsive disorder

^a^Number of the unique pairs identified (e.g., Parents–Offspring, Sibling1–Sibling2)

^b^Number of observations included in the analysis (i.e., all possible combinations of pairs in which members contribute to the analysis with information on OCD and suicide attempts and/or death by suicide: for example, Offspring_(OCD)_−Parents_(Suicide Outcome)_ or Sibling1_(OCD)_–Sibling2_(Suicide outcome)_, Sibling1_(Suicide Outcome)_–Sibling2_(OCD)_)

^c^Probands whose one or both parents died or emigrated from Sweden before 1973 and never returned

^d^Probands with no siblings of a certain degree of relatedness identified from the study cohort

^e^Probands with no full cousins identified from the study cohort or if parents of full cousins are twins

### Sensitivity analyses

Three sets of analyses were performed to check the robustness of our results. First, we explored whether familial coaggregation was better explained by the direct effect of OCD on suicide outcomes within an individual. We re-analyzed each relative cluster after excluding probands with any suicide attempt or a record of death by suicide and relatives with OCD [14, 36, 37]. Second, we examined the extent to which the associations within clusters were explained by comorbidities. For that, we excluded probands and relatives with comorbid psychiatric disorders (one disorder at the time). Finally, we explored whether the results were biased by differential follow-up time and re-run the analyses in the total cohort and in siblings and cousins after excluding those who emigrated or died from reasons other than suicide during the follow-up.

### Quantitative genetic modeling

Separately for each suicide outcome, we measured the contribution of genetic and environmental factors to familial coaggregation with OCD using quantitative genetic modeling [37–39]. Structural equation modeling was used to decompose variance and covariance into additive genetic (A), dominant genetic (D), shared environmental (C), and nonshared environmental (including measurement errors) (E) factors. Given that different types of relatives differentially share etiological factors, we made assumptions of correlation between relatives of A, D, C, and E factors, along the lines of prior studies [32, 40, 41]. In particular, the model fitting was performed under the following assumptions: (i) A factors correlate for full siblings at 0.50 and for half siblings at 0.25, (ii) D factors correlate for full siblings at 0.25 and do not correlate for half siblings (i.e., at 0), (iii) C factors entirely correlate for full siblings and maternal half siblings (i.e., at 1) and do not correlate for paternal half siblings, and (iv) E factors do not correlate for any siblings. The analysis was restricted to full siblings and maternal and paternal half siblings. Within clusters, we randomly selected one pair of siblings from each nuclear family, yielding a total sample of 1,493,534 unique dyads. We assumed that OCD, suicide attempts, and deaths by suicide (as dichotomous variables) represent an underlying multivariate normal distribution of liability and that the observed covariates stem from correlation between the liabilities to OCD and suicide outcomes. To establish which factors out of A, D, C, and E needed to be included in the model to fit, we estimated correlations of OCD with suicide attempts and separately with deaths by suicide within individuals (i.e., phenotypic correlations), between the same trait across siblings (i.e., intraclass correlations), and between trait 1 in one sibling and trait 2 in the other sibling (i.e., cross-sibling cross-trait correlations). We compared the full ADCE model with the reduced submodels ACE, ADE, AE, and CE, using a weighted least squares approach with 95% CIs based on standard errors (Wald CIs). The model with fewer parameters was considered as the best fit, if it was not significantly worse than the full model (by Akaike information criterion and *p* value for the loss of fit for the reduced models). We controlled for sex and year of birth (continuous) of probands and siblings. Preliminary analyses indicated that adjustment for a continuous instead of categorical variable for year of birth did not change the interpretation of associations.

All tests employed two-tailed significance set at *p* < 0.05. Data management was performed using SAS, version 9.4 (SAS Institute, Cary, NC, USA) and analyses were performed using STATA, version 15.1 (StataCorp LLC, College Station, TX, USA), and the package OpenMx [42] in the software R, version 3.4.3 (R Foundation for Statistical Computing, Vienna, Austria).

## Results

### Descriptive statistics

Table 2 presents the characteristics of 3,594,181 cohort members, of which 21,859 were diagnosed with OCD under the study period (57.59% female, *n* = 12,589), corresponding to Kaplan−Meier estimate of expected cumulative incidence at age 46 (i.e., at the end of follow-up) of 1.23% (95% CI 1.21−1.25%), under the assumption of no competing risks. Among individuals with OCD, 2984 had attempted suicide and 159 died by suicide during the follow-up. Nearly 75% of OCD patients had psychiatric comorbidities.

**Table 2:** Distribution of study characteristics in 3,594,181 birth cohort members

Characteristics	Individuals with OCD (*n* = 21,859)^a^	Individuals without OCD (*n* = 3,572,322)	*P*-value^b^
Any suicide attempt, No. (%)	2984 (13.65)	97,037 (2.72)	<0.001
Male	947 (10.22)	45,220 (2.46)	<0.001
Female	2037 (16.18)	51,817 (2.99)	<0.001
Age at first suicide attempt*,* mean (SD), years	23.05 (6.95)	22.60 (7.59)	0.001
Male	24.68 (7.22)	23.76 (7.82)	<0.001
Female	22.30 (6.68)	21.59 (7.23)	<0.001
Death by suicide, No. (%)	159 (0.73)	6746 (0.19)	<0.001
Male	88 (0.95)	4921 (0.27)	<0.001
Female	71 (0.56)	1825 (0.11)	<0.001
Age at death by suicide, mean (SD), years	29.33 (7.09)	26.93 (7.43)	<0.001
Male	29.38 (6.03)	27.02 (7.23)	0.002
Female	29.26 (8.26)	26.69 (7.94)	0.01
Psychiatric comorbidities, No. (%)			
Any comorbidities (at least one)	16,331 (74.71)	390,734 (10.94)	<0.001
Affective disorders	10,101 (46.21)	166,106 (4.65)	<0.001
Anxiety disorders	11,331 (51.84)	150,096 (4.20)	<0.001
Substance use disorders	3465 (15.85)	130,485 (3.65)	<0.001
Psychotic disorders	2189 (9.88)	26,762 (0.75)	<0.001
Personality disorders	3353 (15.34)	32,153 (0.90)	<0.001
Other psychiatric disorders	6416 (29.35)	128,622 (3.60)	<0.001

*OCD* obsessive−compulsive disorder, *SD* standard deviation

^a^Kaplan−Meier estimate of expected cumulative incidence of OCD at age 46 (i.e., at the end of follow-up) is 1.23% (95% CI 1.21−1.25%), under the assumption of no competing risks

^b^*P* values are estimated by chi-square test and Fisher’s exact test for categorical variables and independent-sample two-tailed *t* tests for continuous variables

### Individual and familial risk of suicide outcomes in association with OCD

Individuals with OCD had a significantly increased risk of suicide attempts (OR 5.07, 95% CI 4.88–5.28) and deaths by suicide (OR 4.12, 95% CI 3.52–4.83), compared to individuals without OCD, in sex- and year of birth-adjusted analysis. The risk of suicide attempts was significantly higher in females (OR 5.62, 95% CI 5.36–5.90) than in males (OR 4.23, 95% CI 3.95–4.53) (*p* < 0.001) as well as the risk of death by suicide (females: OR 5.15, 95% CI 4.05–6.53, males: OR 3.57, 95% CI 2.89–4.41; *p* = 0.02).

**Fig. 1:**
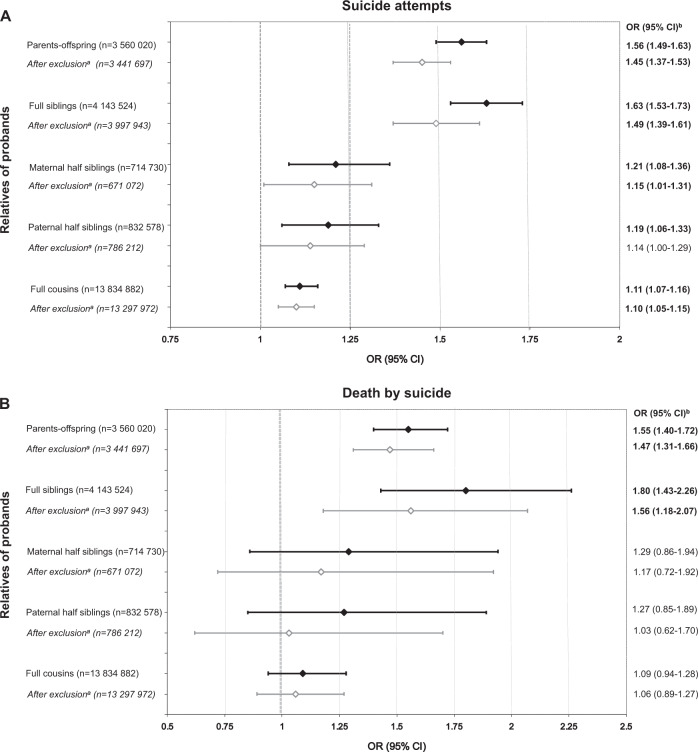
Familial coaggregation between OCD and suicide attempts (panel **a**) and death by suicide (panel **b**) across different types of relatives, before and after excluding probands with any suicidal behavior and relatives with OCD. Note: Numbers in parentheses are the number of observations included in the analysis (i.e., all possible combinations of pairs in which members contribute to the analysis with information on OCD and suicide attempts (panel **a**) or death by suicide (panel **b**). Parents are included in the analysis of “parents−offspring” cluster if none of them have died/emigrated from Sweden (and never returned) prior to 1973. Parents are considered as cases if at least one parent has attempted suicide (panel **a**) or died from suicide (panel **b**). The significant results are highlighted in bold type. ^a^After excluding probands with any suicide attempt or a record of death by suicide and relatives with OCD (the sensitivity analysis on the direct effect of OCD on suicide outcomes within an individual). ^b^Adjusted for sex and birth year (categorized by 10-year increments) of both probands and relatives (only for proband’s sex and year of birth in “parents−offspring” cluster). *OCD* obsessive−compulsive disorder, *OR* odds ratio, *95% CI* 95% confidence interval

Furthermore, an elevated risk of suicide attempts was observed in all relatives of OCD-probands compared to the relatives of probands without OCD, increasing proportionally to the degree of genetic relatedness (Table 3 and Fig. 1a). The risk of death by suicide also increased alongside narrowing genetic distance revealing the same familial pattern as for suicide attempts. However, wider 95% CIs pointed towards an underpowered analysis that was anticipated due to the small number of deaths by suicide (Table 3 and Fig. 1b).

**Table 3:** Familial coaggregation of OCD with suicide attempts and death by suicide across different types of relatives

	Relatives of OCD-probands		Relatives of non-OCD-probands		OR (95%CI)^a^
	Total, no.	Suicide outcome, no. (%)	Total, no.	Suicide outcome, no. (%)	
Suicide attempts					
Parents−offspring	21,593	2112 (9.78)	3,538,427	226,647 (6.41)	**1.56** (**1.49–1.63)**^b^
Full siblings	24,910	1190 (4.78)	4,118,614	115,661 (2.81)	**1.63** (**1.53–1.73)**
Maternal half siblings	5806	359 (6.18)	708,924	36,303 (5.12)	**1.21** (**1.08–1.36)**
Paternal half siblings	6282	338 (5.38)	826,296	38,425 (4.65)	**1.19** (**1.06–1.33)**
Full cousins	85,070	3024 (3.55)	13,749,812	434,302 (3.16)	**1.11** (**1.07–1.16)**
Death by suicide					
Parents−offspring	21,593	365 (1.69)	3,538,427	37,540 (1.06)	**1.55** (**1.40–1.72)**^b^
Full siblings	24,910	79 (0.32)	4,118,614	7066 (0.17)	**1.80** (**1.43–2.26)**
Maternal half siblings	5806	27 (0.47)	708,924	2318 (0.33)	1.29 (0.86–1.94)
Paternal half siblings	6282	24 (0.38)	826,296	2487 (0.30)	1.27 (0.85–1.89)
Full cousins	85,070	172 (0.20)	13,749,812	24,912 (0.18)	1.09 (0.94–1.28)

Note: Parents are included in the analysis of “parents−offspring” cluster if none of them have died/emigrated from Sweden (and never returned) prior to 1973. Parents are considered as cases for suicide behavior if at least one parent has a corresponding outcome, i.e., attempted suicide or died from suicide. The significant results are highlighted in bold type

*OCD* obsessive−compulsive disorder, *OR* odds ratio, *95% CI* 95% confidence interval

^a^Adjusted for sex and birth year (categorized by 10-year increments) of both probands and relatives

^b^Adjusted for sex and birth year (categorized by 10-year increments) of the probands

### Sensitivity analyses

When we excluded relatives with OCD and probands with any suicidal outcomes from the analysis of OCD and suicide attempts, all ORs were slightly attenuated, yet remained significant, except for paternal half siblings (Fig. 1a and Supplementary Table 2). For deaths by suicide, the ORs diminished in all relative clusters, yet the increased risks among parents and full siblings remained significant (Fig. 1b and Supplementary Table 2).

When probands and relatives with psychiatric comorbidities were excluded, the risks for OCD and suicide attempts, as well as for OCD and death by suicide, were attenuated, although the patterns of risk across relatives remained mainly the same (Supplementary Table 3).

Finally, our effort to overcome the follow-up bias by excluding all migrations and deaths did not alter any previous results (Supplementary Table 4).

### Quantitative genetic modeling

Table 4 reports sex- and year of birth-adjusted phenotypic, intraclass, and cross-trait cross-sibling correlations of OCD with suicide outcomes. The phenotypic correlation between OCD and suicide attempts within individuals was 0.30 (95% CI 0.29–0.31). Intraclass and cross-trait cross-sibling correlations were higher in full siblings than in maternal half siblings. Cross-trait cross-sibling correlations were similar in maternal and paternal half siblings. The ACE submodel provided the best fit (Supplementary Table 5). As a result, 60.7% (95% CI 32.1–89.4) of phenotypic correlation between OCD and suicide attempts appeared to be explained by additive genetics and 40.4% (95% CI 24.2–56.6) were attributable to nonshared environment. The contribution of shared environment was negligible (−1.2%; 95% CI −14.8 to 12.5).

**Table 4:** Phenotypic, intraclass (ICC), and cross-trait cross-sibling (CTCS) correlations between OCD and suicide attempts and death by suicide, presented with 95% confidence intervals and adjusted for sex and year of birth (continuous) of probands and relatives

	No. of unique dyads (one per nuclear family)	No. of concordant pairs	Phenotypic correlation	ICC for OCD	ICC for suicidal behavior outcome	CTCS correlation for OCD and outcome
Suicide attempts						
Full cohort	1,493,534	922^a^	**0.30** (**0.29–0.31)**	NA	NA	NA
Full siblings	1,163,294	613^a^	**0.30** (**0.29–0.31)**	**0.24 (0.22–0.26)**	**0.29 (0.28–0.30)**	**0.09 (0.07–0.10)**
Maternal half siblings	164,070	162^a^	**0.30** (**0.28–0.32)**	**0.11 (0.05–0.18)**	**0.16 (0.14–0.18)**	**0.05 (0.02–0.08)**
Paternal half siblings	166,170	147^a^	**0.30** (**0.28–0.32)**	0.06 (−0.01 to 0.14)	**0.11 (0.08–0.13)**	**0.05 (0.02–0.08)**
Death by suicide						
Full cohort	1,493,534	60^b^	**0.19** (**0.16–0.21)**	NA	NA	NA
Full siblings	1,163,294	42^b^	**0.20** (**0.17–0.23)**	**0.24 (0.21–0.26)**	**0.24 (0.18–0.29)**	**0.07 (0.03–0.10)**
Maternal half siblings	164,070	9^b^	**0.11** (**0.04–0.18)**	**0.11 (0.05–0.18)**	0.01 (−0.16 to 0.18)	0.01 (−0.08 to 0.09)
Paternal half siblings	166,170	9^b^	**0.19** (**0.13–0.25)**	0.07 (−0.01 to 0.14)	0.04 (−0.13 to 0.21)	0.03 (−0.06 to 0.11)

Note: The 95% confidence intervals are presented in parentheses. The significant results are highlighted in bold type

*CTCS* cross-trait cross-sibling, *ICC* intraclass correlation, *NA* not applicable, *OCD* obsessive−compulsive disorder

^a^Number of sibling pairs where one sibling had a diagnosis of OCD and another sibling had a record of suicide attempt. In full siblings, five pairs were doubly concordant (i.e., both siblings had both OCD and suicide attempts). For maternal half siblings, one pair was doubly concordant. In paternal half siblings, no pair was doubly concordant

^b^Number of sibling pairs where one sibling had a diagnosis of OCD and another sibling had a record of death by suicide. No sibling pair was doubly concordant (i.e., in no pair both siblings had OCD and died by suicide)

The phenotypic correlation between OCD and death by suicide was 0.19 (95% CI 0.16–0.21) (Table 4). All correlations in maternal half siblings were less than half of that in full siblings, while cross-trait cross-sibling correlations were higher in paternal than in maternal half siblings. AE submodel provided the best fit (Supplementary Table 6). The phenotypic correlation between OCD and death by suicide was explained by additive genetics (65.8%; 95% CI 25.9–105.6) and nonshared environment (34.2%; 95% CI −5.6 to 74.1). However, the analyses were underpowered, with the full ADCE model being numerically unstable.

## Discussion

This population-based birth cohort study is the first to explore familial coaggregation of OCD and suicide outcomes and to quantify the contribution of genetic and environmental factors to familial liability. Four main findings emerged. First, confirming previous studies conducted in clinical and community settings [1–5, 43–46], individuals with OCD were at significantly increased risk of suicide attempts and death by suicide compared to individuals without OCD, and the risks were more pronounced for female than male OCD sufferers, similar to the results of another Swedish study on OCD and suicide [2]. This was anticipated in relation to suicide attempts, as nonfatal suicidal behavior is known to be more common in females [28, 47]. A corresponding higher risk for death by suicide can stem from relatively similar proportions of females and males who died by suicide in the OCD group, while in the general population, females were considerably less likely to die by suicide, compared to males [28, 47]. Overall, our estimates for death by suicide likely represent an underestimation of the actual risk due to the young age of the cohort. Indeed, in a previous Swedish register study [2], the mean age at death by suicide was 42.5 years (vs. 29.3 years in the current study), resulting in an OR of 9.83 (vs. 4.12 in the current study). Despite the fact that death by suicide is relatively uncommon among OCD sufferers (0.73% in OCD patients and 0.19% in individuals without OCD), the reported proportions of suicide attempts (13.65% in OCD patients and 2.72% in individuals without OCD), together with previous findings, challenge the traditional view of OCD as a low risk disorder for suicidal behavior.

Second, the patterns of risk of suicide attempts and deaths by suicide that appeared among the relatives of OCD-probands, with the strength of associations increasing proportionally to the degree of genetic relatedness, suggest that familial risk factors underlie, at least partly, an association between OCD and suicide outcomes. A contribution of genetic factors was further supported by the risk of suicide attempts being higher in full siblings compared to maternal half siblings as these types of relatives share a substantial amount of environment as they grow up but differ on the proportion of shared cosegregating alleles (50% vs. 25%, respectively). A minor potential role of familial environment was apparent from the rather negligible difference in risks observed in maternal and paternal half siblings given that these relative categories both share 25% of additive genetics, but maternal half siblings tend to share more environmental factors. An underpowered analysis of coaggregation between OCD and death by suicide precluded us from drawing definite conclusions, although the higher risk of death by suicide in full siblings, compared to maternal half siblings, suggests a possible contribution of genetic factors. A sensitivity analysis further supported the familial coaggregation of OCD with suicide outcomes, rather than the direct effect of OCD on suicidal behavior within an individual.

Third, quantitative genetic modeling confirmed observations from the family data to indicate that genetic factors largely explain the phenotypic cross-disorder correlations, though the contribution of genetic factors is slightly more pronounced for coaggregation of OCD and death by suicide (66%) than that of OCD and suicide attempts (60%). With samples of genotyped individuals with OCD and suicidal behavior increasing worldwide, it is expected that the current results will be replicated and confirmed at the molecular genetic level. If biological pleiotropy is demonstrated, one implication for future research is that identification of genes implicated in OCD may theoretically also shed light on genetic variants that increase risk of suicide, and vice versa.

Fourth, in our study, a smaller but substantial part of familial coaggregation was attributed to nonshared environment (34% for OCD and death by suicide and 40% for OCD and suicide attempts). This suggests that suicidal behavior can also be partially conceptualized as a functional consequence of OCD, a chronic disorder that substantially impairs the person’s quality of life [48, 49] and ability to fully participate in society [50, 51]. This is a particularly important finding since a deeper knowledge of these unique risk factors has the potential to improve identification of OCD patients at high risk of suicidal behaviors and provide a window of opportunity for the design and implementation of targeted preventive interventions in individuals at-risk. In addition, unique environmental factors, if modifiable, may serve as intervention targets themselves and thus be “proxy” targets for suicide prevention in OCD patients (e.g., treatment of comorbid psychiatric comorbidities, increase of social support). Taken together, our findings warrant further research into potential evocative gene−environment correlations where genetically influenced behavior (i.e., OCD) may in turn influence the environment [48–53] in a way that the risk of suicide is increased [28, 54–58] (e.g., social isolation, academic underachievement, unemployment, low socioeconomic level, being unmarried, poor quality of life).

### Strengths and limitations

Along with a large study size, the strengths of the study include the use of nationwide Swedish registers with prospective and uniformed data collection which minimizes the risk of selection, recall, and report biases. The completeness of the MGR [17] ensured ascertainment of a large sample of relatives with different shares of common genetic and environmental factors that allowed an assessment of familial coaggregation in distinct relative clusters. Furthermore, our definitions of clinical cases have previously been validated for Swedish data on OCD [21], many psychiatric comorbidities [59–62], and death by suicide [25, 63], which decreases diagnostic misclassification. The findings were largely unchanged in various sensitivity analyses where psychiatric comorbidities were taken into account.

Several limitations should also be considered. First, the study cohort does not represent the totality of individuals in Sweden with OCD or suicide attempts due to a number of reasons: (i) not all OCD sufferers seek help [64–66] and low rates of healthcare utilization are known for suicide attempts due to the sensitive nature of these behaviors and given that not all self-harm cases need inpatient or specialized outpatient treatment [67, 68]; (ii) the coverage of the NPR is incomplete since prior to 2001 only hospital admissions were registered; and (iii) the NPR does not include diagnostic information from primary care services or nonmedical specialists [18]. Second, compared to the higher validity of ICD-10 codes for OCD, the validity of ICD-8 and ICD-9 codes is slightly lower [19]. While this should not be crucial for our cohort members with only 1.53% of OCD cases never (re-)appearing in ICD-10, it may affect the collection of parental OCD used in the sensitivity analysis. Third, data on biological paternity in the MGR are self-reported by the mother and can be inaccurate. However, in an international review [69], paternal discrepancy was reported to be low (median 3.7%). Fourth, despite a large study size, our analyses were underpowered for capturing associations between OCD and death by suicide. Small number of cases also affected the stability of the models in quantitative genetic analyses. Fifth, out of the factors that are potentially shared between OCD and suicidal behavior and can explain their familial coaggregation, we only controlled for the impact of psychiatric comorbidities. Due to data unavailability, we could not explore the impact of, for example, childhood trauma (i.e., abuse and neglect), which is thought to predispose individuals for the development of both OCD and suicidal behavior [70, 71]. Finally, a tendency to joint physical custody, that currently applies to 30% of Swedish children after parental separation [72], might have diminished the difference in shared environment between maternal and paternal half siblings.

### Conclusions

The relation between OCD and suicidal behavior appears to be complex. Although genetic factors largely underlie familial coaggregation between OCD and both outcomes, the contribution of the unique nonshared environment is also considerable and suggests an opportunity for prevention and intervention in high-risk individuals.

## Supplementary information

Supplementary material

Supplementary Table 1

Supplementary Table 2

Supplementary Table 3

Supplementary Table 4

Supplementary Table 5

Supplementary Table 6
