# Supplementary material for: Genetic and environmental sources of familial coaggregation of obsessive−compulsive disorder and suicidal behavior: a population-based birth cohort and family study
Source: Mol Psychiatry. 2019 Apr 8;26(3):974–85. doi: 10.1038/s41380-019-0417-1 (PMC7910213; doi:10.1038/s41380-019-0417-1)
Supplement: Supplementary file 1 — Supplementary material [file 41380_2019_417_MOESM1_ESM.docx]

**Supplementary material for:**

*Genetic and environmental sources of familial coaggregation of obsessive-compulsive disorder and suicidal behavior: A population-based birth cohort and family study*

*Anna Sidorchuk, PhD^1#^, Ralf Kuja-Halkola, PhD^2^, Bo Runeson, PhD^3^, Paul Lichtenstein, PhD^2^, Henrik Larsson, PhD^2,4^, Christian Rück, PhD^1^, Brian M. D’Onofrio PhD^2,5^, David Mataix-Cols PhD^1^, Lorena Fernández de la Cruz, PhD^1^*

^1^ Centre for Psychiatry Research, Department of Clinical Neuroscience, Karolinska Institutet, & Stockholm Health Care Services, Stockholm County Council, Stockholm, Sweden

^2^ Department of Medical Epidemiology and Biostatistics, Karolinska Institutet, Stockholm, Sweden

^3^ Centre for Psychiatry Research, Department of Clinical Neuroscience, Karolinska Institutet, & Stockholm Health Care Services, Stockholm County Council, S:t Görans Hospital, SE-112 61, Stockholm, Sweden

^4^ School of Medical Sciences, Örebro University, Örebro, Sweden

^5^ Department of Psychological and Brain Science, Indiana University, Bloomington, IN, USA

^#^ Corresponding author: Anna Sidorchuk, MD, PhD, Centre for Psychiatry Research, Department of Clinical Neuroscience, Karolinska Institutet, & Stockholm Health Care Services, Stockholm County Council, Gävlegatan 22B, 113 30 Stockholm, Sweden, [anna.sidorchuk@ki.se](mailto:anna.sidorchuk@ki.se)

**Additional information on the Swedish National Registers**

*The Multi-Generation Register (MGR)* contains information on biological and adopted parents of all individuals who was born in Sweden from 1932 onwards or has ever been registered in the country since 1961 [1]. With the mother as informant, the father is defined as the mother’s husband at the time of birth or the man acknowledged as the father by unmarried mothers. The MGR spans over five generations and contains data on 100% of mothers and 98% of fathers for those born in Sweden since 1961 that enables constructing family pedigrees of different genetic and environmental distances [2]. *The National Patient Register* captures diagnostic information from somatic and psychiatric inpatient care (covered since 1969 and 1973, respectively) and specialist outpatient care (since 2001), based on the Swedish version of the International Classification of Diseases, Eighth Revision (ICD-8) (1969-1986), ICD-9 (1987-1996), and ICD-10 (1997-onwards) [3]. *The Cause of Death Register* includes information on all deaths of Swedish residents, occurring in Sweden or abroad, with dates and the international version of the ICD codes for underlying and contributory causes of deaths since 1952 [4]. *The Total Population Register* records demographic data of all Swedish inhabitants since 1968, and *the Migration Register* captures migration in and out of Sweden [5].

**References**

1. Ekbom A. The Swedish Multi-generation register. *Methods Mol Biol* 2011;675:215-220.

2. Statistics Sweden. Multi-generation register 2016 - A description of contents and quality. Background Facts, Population and Welfare Statistics, 2017. https://www.scb.se/contentassets/95935956ea2b4fa9bcaab51afa259981/ov9999_2016a01_br_be96br1702eng.pdf, Accessed December 3, 2018.

3. Ludvigsson JF, Andersson E, Ekbom A, Feychting M, Kim JL, Reuterwall C, et al. External review and validation of the Swedish national inpatient register. *BMC Public Health* 2011;11:450.

4. Brooke HL, Talback M, Hornblad J, Johansson LA, Ludvigsson JF, Druid H, et al. The Swedish cause of death register. *Eur J Epidemiol* 2017;32(9):765-773.

5. Ludvigsson JF, Almqvist C, Bonamy AK, Ljung R, Michaelsson K, Neovius M, et al. Registers of the Swedish total population and their use in medical research. *Eur J Epidemiol* 2016;31(2):125-136.
