# Supplementary material for: Genetic and environmental sources of familial coaggregation of obsessive−compulsive disorder and suicidal behavior: a population-based birth cohort and family study
Source: Mol Psychiatry. 2019 Apr 8;26(3):974–85. doi: 10.1038/s41380-019-0417-1 (PMC7910213; doi:10.1038/s41380-019-0417-1)
Supplement: Supplementary file 2 — Supplementary Table 1 [file 41380_2019_417_MOESM2_ESM.docx]

**Supplementary Table 1.** International Classification of Disease (ICD) codes for obsessive-compulsive disorder, suicide attempt, death by suicide, and comorbid psychiatric disorders in ICD-8, ICD-9, and ICD-10

| **Diagnoses** | **ICD-8** | **ICD-9** | **ICD-10** |
| --- | --- | --- | --- |
| Obsessive-compulsive disorder^a^ | 300.3 | 300D | F42 |
| ***Suicide attempts^a^ (death by suicide)^b^*** | |  |  |
| Suicide and self-inflicted injury | E950-E959 | E950-E959 |  |
| Injury undetermined whether accidental or purposely inflicted | E980-E989 | E980-E989 |  |
| Intentional self-harm |  |  | X60-X84 |
| Events of undetermined intent |  |  | Y10-Y34 |
| ***Comorbid psychiatric disorders^a^*** | |  |  |
| Affective disorders | 296.0, 296.1, 296.2, 296.3, 296.88, 300.4 | 296A-C, 296E, 296W, 300E | F30-F39, except F32.3 |
| Anxiety disorders | 300.0, 300.2 | 300A, 300C | F40, F41 |
| Personality disorders | 301 | 301 | F60 |
| Substance use disorders | 303, 304 | 303, 304, 305A, 305X | F10-F19, except F1x.5 |
| Psychotic disorders | 291, 295, 296.99, 297, 298, 299 | 291, 292, 295, 296D, 296X, 297, 298, 299 | F20-F25, F28, F29, F32.3, x.5 in F10-F19 |
| ‘Other’ psychiatric disorders^c^ | 300.1, 300.5, 300.6, 300.7, 300.88, 300.99, 307 | 300B, 300F, 300G, 300H, 300W, 300X, 307B, 307F, 309 | F43-F45, F48, F50 |

^a^ Diagnoses of obsessive-compulsive disorder, suicide attempts, and comorbid psychiatric disorders were retrieved from the National Patient Register and were coded according to the Swedish version of ICD-codes of 8^th^/9^th^/10^th^ revisions

^b^ Diagnoses of death by suicide were retrieved from the Cause of Death Register using the same codes as for suicide attempts and were coded according to the International version of ICD-codes in its 8^th^, 9^th^, or 10^th^ revision

^c^ Include reaction for severe stress, adjustment, dissociative, somatoform and other neurotic disorders
