# Supplementary material for: Genetic and environmental sources of familial coaggregation of obsessive−compulsive disorder and suicidal behavior: a population-based birth cohort and family study
Source: Mol Psychiatry. 2019 Apr 8;26(3):974–85. doi: 10.1038/s41380-019-0417-1 (PMC7910213; doi:10.1038/s41380-019-0417-1)
Supplement: Supplementary file 3 — Supplementary Table 2 [file 41380_2019_417_MOESM3_ESM.docx]

**Supplementary Table 2.** Sensitivity analysis. Familial co-aggregation of OCD with suicide attempts and death by suicide across different types of relatives after excluding probands with the records of any suicidal behavior (suicide attempts or death by suicide) and relatives with OCD

|  | **Relatives of OCD probands** | | **Relatives of non-OCD probands** | | **OR (95%CI)^a^** |
| --- | --- | --- | --- | --- | --- |
|  | **Total, no.** | **Suicide outcome,**  **no. (%)** | **Total, no.** | **Suicide outcome,**  **no. (%)** |  |
| ***Suicide attempt*** |  |  |  |  |  |
| Parents-offspring | 18 265 | 1561 (8.55) | 3 423 432 | 205 340 (6.00) | **1.45 (1.37-1.53)**^b^ |
| Full siblings | 20 769 | 819 (3.94) | 3 977 174 | 100 699 (2.53) | **1.49 (1.39-1.61)** |
| Maternal half siblings | 4533 | 248 (5.47) | 666 539 | 31 931 (4.79) | **1.15 (1.01-1.31)** |
| Paternal half siblings | 4995 | 245 (4.90) | 781 217 | 34 561 (4.42) | 1.14 (1.00-1.29) |
| Full cousins | 72 115 | 2394 (3.32) | 13 225 857 | 395 806 (2.99) | **1.10 (1.05-1.15)** |
| ***Death by suicide*** |  |  |  |  |  |
| Parents-offspring | 18 265 | 276 (1.51) | 3 423 432 | 34 597 (1.01) | **1.47 (1.31-1.66)**^b^ |
| Full siblings | 20 769 | 52 (0.25) | 3 977 174 | 6318 (0.16) | **1.56 (1.18-2.07)** |
| Maternal half siblings | 4533 | 18 (0.40) | 666 539 | 2067 (0.31) | 1.17 (0.72-1.92) |
| Paternal half siblings | 4995 | 15 (0.30) | 781 217 | 2278 (0.29) | 1.03 (0.62-1.70) |
| Full cousins | 72 115 | 137 (0.19) | 13 225 857 | 23 321 (0.18) | 1.06 (0.89-1.27) |

*Note*: Parents are included in the analysis of ‘parents-offspring’ cluster if none of them have died / emigrated from Sweden (and never returned) prior to 1973. Parents are considered as cases for suicide behavior if at least one parent has a corresponding outcome, i.e., attempted suicide or died from suicide. The significant results are highlighted in bold type.

^a^ Adjusted for sex and birth year (categorized by 10-year increments) of both probands and relatives

^b^ Adjusted for sex and birth year (categorized by 10-year increments) of the probands

Abbreviations: OCD, obsessive-compulsive disorder; OR, odds ratio; 95%CI, the 95% confidence intervals
