# Supplementary material for: Genetic and environmental sources of familial coaggregation of obsessive−compulsive disorder and suicidal behavior: a population-based birth cohort and family study
Source: Mol Psychiatry. 2019 Apr 8;26(3):974–85. doi: 10.1038/s41380-019-0417-1 (PMC7910213; doi:10.1038/s41380-019-0417-1)
Supplement: Supplementary file 4 — Supplementary Table 3 [file 41380_2019_417_MOESM4_ESM.docx]

**Supplementary Table 3.** Sensitivity analysis. Familial co-aggregation of OCD with suicide attempts and death by suicide across different types of relatives after excluding probands and relatives with comorbid psychiatric disorders

|  | **OR (95% CI)^a^** | | | | | | |
| --- | --- | --- | --- | --- | --- | --- | --- |
|  | **Before exclusion** | **Excluding affective disorders** | **Excluding**  **anxiety**  **disorders** | **Excluding personality disorders** | **Excluding psychotic disorders** | **Excluding substance use disorders** | **Excluding ‘other’ psychiatric comorbidities** |
| ***Suicide attempt*** |  |  |  |  |  |  |  |
| Parents-offspring^b^ | **1.56 (1.49-1.63)** | **1.21 (1.11-1.33)** | **1.28 (1.18-1.40)** | **1.40 (1.32-1.48)** | **1.47 (1.39-1.55)** | **1.36 (1.27-1.46)** | **1.33 (1.24-1.43)** |
| Full siblings | **1.63 (1.53-1.73)** | **1.21 (1.07-1.37)** | **1.25 (1.10-1.41)** | **1.43 (1.32-1.54)** | **1.57 (1.47-1.68)** | **1.40 (1.25-1.48)** | **1.42 (1.30-1.56)** |
| Maternal half-siblings | **1.21 (1.08-1.36)** | **1.29 (1.05-1.58)** | **1.29 (1.04-1.59)** | **1.19 (1.04-1.37)** | **1.24 (1.10-1.40)** | **1.22 (1.03-1.44)** | **1.21 (1.03-1.43)** |
| Paternal half-siblings | **1.19 (1.06-1.33)** | 1.04 (0.85-1.29) | 1.02 (0.82-1.28) | 1.12 (0.98-1.29) | **1.18 (1.04-1.73)** | 1.00 (0.84-1.19) | 1.17 (0.99-1.38) |
| Full cousins | **1.11 (1.07-1.16)** | 1.01 (0.94-1.08) | **1.09 (1.01-1.16)** | **1.06 (1.01-1.11)** | **1.12 (1.07-1.17)** | **1.08 (1.02-1.14)** | **1.07 (1.02-1.13)** |
| ***Death by suicide*** |  |  |  |  |  |  |  |
| Parents-offspring^b^ | **1.55 (1.40-1.72)** | **1.25 (1.01-1.55)** | **1.46 (1.22-1.75)** | **1.45 (1.27-1.65)** | **1.42 (1.24-1.60)** | **1.49 (1.28-1.74)** | **1.53 (1.31-1.79)** |
| Full siblings | **1.80 (1.43-2.26)** | 1.36 (0.85-2.18) | **1.87 (1.28-2.72)** | **1.53 (1.14-2.06)** | **1.70 (1.30-2.23)** | **1.47 (1.05-2.05)** | 1.26 (0.88-1.82) |
| Maternal half-siblings | 1.29 (0.86-1.94) | 0.88 (0.40-1.97) | 0.99 (0.45-2.20) | 0.96 (0.56-1.67) | 1.27 (0.79-2.06) | 0.34 (0.11-1.06) | 0.99 (0.53-1.84) |
| Paternal half-siblings | 1.27 (0.85-1.89) | 0.85 (0.38-1.89) | 0.63 (0.24-1.69) | 0.94 (0.55-1.66) | 1.04 (0.63-1.73) | 1.04 (0.54-1.99) | 0.70 (0.33-1.47) |
| Full cousins | 1.09 (0.94-1.28) | 1.05 (0.81-1.37) | 1.13 (0.86-1.48) | 1.03 (0.85-1.26) | 1.08 (0.90-1.29) | 1.06 (0.85-1.31) | 0.98 (0.78-1.23) |

*Note*: Parents are included in the analysis of ‘parents-offspring’ cluster if none of them have died / emigrated from Sweden (and never returned) prior to 1973. Parents are considered as cases for suicide behavior if at least one parent has a corresponding outcome (i.e., attempted suicide or died from suicide), and as cases for comorbid psychiatric disorders if at least one parent has a corresponding disorder. The significant results are highlighted in bold type.

^a^ Adjusted for sex and birth year (categorized by 10-year increments) of both probands and relatives

^b^ Adjusted for sex and birth year (categorized by 10-year increments) of the probands

Abbreviations: OCD, obsessive-compulsive disorder; OR, odds ratio; 95%CI, the 95% confidence intervals
