# Supplementary material for: Genetic and environmental sources of familial coaggregation of obsessive−compulsive disorder and suicidal behavior: a population-based birth cohort and family study
Source: Mol Psychiatry. 2019 Apr 8;26(3):974–85. doi: 10.1038/s41380-019-0417-1 (PMC7910213; doi:10.1038/s41380-019-0417-1)
Supplement: Supplementary file 5 — Supplementary Table 4 [file 41380_2019_417_MOESM5_ESM.docx]

**Supplementary Table 4.** Sensitivity analysis. Familial co-aggregation of OCD with suicide attempts and death by suicide across siblings and cousins in a restricted cohort, i.e., after excluding probands and relatives who emigrated or died from the reasons other than suicide during the follow-up

|  | **Relatives of OCD probands** | | **Relatives of non-OCD probands** | | **OR (95%CI)^a^** |
| --- | --- | --- | --- | --- | --- |
|  | **Total, no.** | **Suicide outcome,**  **no. (%)** | **Total, no.** | **Suicide outcome,**  **no. (%)** |  |
| ***Suicide attempt*** |  |  |  |  |  |
| Full siblings | 22 906 | 1096 (4.78) | 3 688 356 | 103 346 (2.80) | **1.62 (1.52-1.73)** |
| Maternal half-siblings | 5305 | 329 (6.20) | 637 807 | 32 333 (5.07) | **1.23 (1.09-1.28)** |
| Paternal half-siblings | 5672 | 302 (5.32) | 723 014 | 33 614 (4.65) | **1.18 (1.04-1.32)** |
| Full cousins | 85 070 | 3024 (3.55) | 13 749 812 | 434 302 (3.16) | **1.11 (1.07-1.16)** |
| ***Death by suicide*** |  |  |  |  |  |
| Full siblings | 22 906 | 75 (0.33) | 3 688 356 | 6413 (0.17) | **1.81 (1.43-2.28)** |
| Maternal half-siblings | 5305 | 27 (0.51) | 637 807 | 2099 (0.33) | 1.41 (0.94-2.12) |
| Paternal half-siblings | 5672 | 22 (0.39) | 723 014 | 2231 (0.31) | 1.25 (0.83-1.90) |
| Full cousins | 85 070 | 172 (0.20) | 13 749 812 | 24 912 (0.18) | 1.09 (0.94-1.28) |

*Note*: The significant results are described in bold type.

^a^ Adjusted for sex and birth year (categorized by 10-year increments) of both probands and relatives

Abbreviations: OCD, obsessive-compulsive disorder; OR, odds ratio; 95%CI, the 95% confidence intervals
