# Supplementary material for: Genetic and environmental sources of familial coaggregation of obsessive−compulsive disorder and suicidal behavior: a population-based birth cohort and family study
Source: Mol Psychiatry. 2019 Apr 8;26(3):974–85. doi: 10.1038/s41380-019-0417-1 (PMC7910213; doi:10.1038/s41380-019-0417-1)
Supplement: Supplementary file 6 — Supplementary Table 5 [file 41380_2019_417_MOESM6_ESM.docx]

**Supplementary Table 5.** Quantitative genetic modelling. Genetic and environmental parameter estimates for familial co-aggregation of OCD and suicide attempts

| **Parameters** | **Models^a^** | | | | |
| --- | --- | --- | --- | --- | --- |
|  | **ADCE** | **ACE^b^** | **ADE** | **AE** | **CE** |
| ***Model fitting^c^*** |  |  |  |  |  |
| No. of parameters | 38 | **35** | 35 | 32 | 32 |
| AIC | 4206.72 | **4205.92** | 4214.84 | 4213.00 | 4454.23 |
| p-value | na | **0.166** | 0.003 | 0.007 | <0.001 |
| ***OCD, % (95% CI)*** |  |  |  |  |  |
| OCD due to A | 25.6 (-3.8, 55.0) | **37.9 (17.7, 58.1)** | 37.0 (17.4, 56.5) | 47.3 (42.6, 51.9) | 0.0 (0.0, 0.0) |
| OCD due to D | 23.7 (-16.7, 64.2) | **0.0 (0.0, 0.0)** | 21.8 (-18.4, 62.0) | 0.0 (0.0, 0.0) | 0.0 (0.0, 0.0) |
| OCD due to H | 49.3 (21.5, 77.1) | **37.9 (17.7, 58.1)** | 58.7 (37.0, 80.5) | 47.3 (42.6, 51.9) | 0.0 (0.0, 0.0) |
| OCD due to C | 5.2 (-4.6, 15.0) | **4.6 (-5.1, 14.4)** | 0.0 (0.0, 0.0) | 0.0 (0.0, 0.0) | 22.5 (20.2, 24.7) |
| OCD due to E | 45.5 (22.5, 68.5) | **57.4 (46.5, 68.4)** | 41.3 (19.5, 63.0) | 52.7 (48.1, 57.4) | 77.5 (75.3, 79.8) |
| ***Suicide attempts, % (95% CI)*** |  |  |  |  |  |
| Suicide attempts due to A | 42.4 (33.8, 51.1) | **47.8 (41.6, 53.9)** | 53.8 (47.9, 59.6) | 58.0 (56.3, 59.7) | 0.0 (0.0, 0.0) |
| Suicide attempts due to D | 10.9 (-1.4, 23.2) | **0.0 (0.0, 0.0)** | 9.3 (-3.0, 21.6) | 0.0 (0.0, 0.0) | 0.0 (0.0, 0.0) |
| Suicide attempts due to H | 53.3 (44.5, 62.1) | **47.8 (41.6, 53.9)** | 63.1 (56.1, 70.0) | 58.0 (56.3, 59.7) | 0.0 (0.0, 0.0) |
| Suicide attempts due to C | 5.3 (2.3, 8.2) | **5.1 (2.2, 8.0)** | 0.0 (0.0, 0.0) | 0.0 (0.0, 0.0) | 26.9 (26.1, 27.7) |
| Suicide attempts due to E | 41.4 (34.1, 48.8) | **47.2 (43.7, 50.6)** | 36.9 (30.0, 43.9) | 42.0 (40.3, 43.7) | 73.1 (72.3, 73.9) |
| ***Bivariate explained covariance, % (95% CI)*** | |  |  |  |  |
| Covariance due to A | 69.1 (30.0, 108.2) | **60.7 (32.1, 89.4)** | 67.2 (39.9, 94.4) | 58.4 (49.8, 67.19 | 0.0 (0.0, 0.0) |
| Covariance due to D | -18.9 (-76.4, 38.5) | **0.0 (0.0, 0.0)** | -19.5 (-76.9, 37.9) | 0.0 (0.0, 0.0) | 0.0 (0.0, 0.0) |
| Covariance due to H | 50.2 (8.1, 92.3) | **60.7 (32.1, 89.4)** | 47.7 (15.0, 80.4) | 58.4 (49.8, 67.1) | 0.0 (0.0, 0.0) |
| Covariance due to C | -1.1 (-14.7, 12.5) | **-1.2 (-14.8, 12.5)** | 0.0 (0.0, 0.0) | 0.0 (0.0, 0.0) | 26.5 (22.4, 30.6) |
| Covariance due to E | 50.9 (15.8, 86.1) | **40.4 (24.2, 56.6)** | 52.3 (19.6, 85.0) | 41.6 (32.9, 50.2) | 73.5 (69.4, 77.6) |
| ***Bivariate correlations, % (95% CI)*** | |  |  |  |  |
| rA | 0.63 (0.15, 1.11) | **0.43 (0.20, 0.65)** | 0.45 (0.24, 0.66) | 0.33 (0.28, 0.38) | na |
| rD | -0.35 (-1.52, 0.81) | **na** | -0.41 (-1.75, 0.93) | na | na |
| rH | 0.29 (0.04, 0.55) | **0.43 (0.20, 0.65)** | 0.23 (0.07, 0.40) | 0.33 (0.28, 0.38) | na |
| rC | -0.06 (-0.85, 0.72) | **-0.07 (-0.92, 0.78)** | Na | na | 0.32 80.27, 0.37) |
| rE | 0.35 (0.10, 0.60) | **0.23 (0.14, 0.33)** | 0.40 (0.14, 0.67) | 0.26 (0.21, 0.32) | 0.29 (0.27, 0.31) |

*Note:* The comparison of the full model (ADCE model) with the reduced submodels ACE, ADE, AE, and CE was performed by using a weighted least squares approach with the 95% confidence intervals (CI) based on standard errors (Wald CI). Therefore, the 95% CIs might fall above 1 or below -1 for correlations, and below 0 for variance components.

^a^ Adjusted for sex and birth year (continuous) of both probands and relatives

^b^ Best fitting model – ACE – includes additive genetic, shared environment and non-shared environment parameters (i.e., without a significant decrease in a fit after dropping dominant genetic parameter from the full ADCE model)

^c^ Model fitting assumptions were: (i) additive genetic factors correlate for full siblings at 0.50 and for half siblings at 0.25, (ii) dominant genetic factors correlate for full siblings at 0.25 and for half siblings at 0, (iii) shared environmental factors correlate for full siblings and maternal half siblings at 1 and for paternal half siblings at 0, and (iv) non-shared environmental factors do not to correlate between siblings

Abbreviations: AIC, Akaike information criterion; p-value, for the loss of fit for the reduced models; A, additive genetic parameter; D, dominant genetic parameter; H, combined additive and dominant genetic parameters (A+D); C, shared environmental parameter; E, non-shared environmental parameter (including measurement errors); OCD, obsessive-compulsive disorder; rA, additive genetic correlation; rD dominant genetic correlation; rH combined A and D correlation; rC, shared environmental correlation; rE, non-shared environmental correlation, na, not applicable
